# Ifosfamide‐induced acute kidney injury in a patient with leiomyosarcoma: A case report

**DOI:** 10.1002/cnr2.1666

**Published:** 2022-07-13

**Authors:** Javad Boskabadi, Ehsan Yousefi‐Mazhin, Ebrahim Salehifar

**Affiliations:** ^1^ Student Research Committee, Pharmaceutical Sciences Research Center, Hemoglobinopathy Institute, Department of Clinical Pharmacy Mazandaran University of Medical Sciences Sari Iran; ^2^ Pharmaceutical Sciences Research Center, Hemoglobinopathy Institute, Department of Clinical Pharmacy Mazandaran University of Medical Sciences Sari Iran

**Keywords:** acute kidney injury, ifosfamide, leiomyosarcoma, mesna, N‐acetylcysteine

## Abstract

**Background:**

Leiomyosarcoma (LMS) is an aggressive soft tissue sarcoma that is derived from smooth muscles. Ifosfamide is in use for advanced metastatic LMS.

**Case:**

A‐44‐years old woman with a chief complaint of pain in the epigastric area, itching, coughing, nausea, and vomiting was referred to the emergency department. Her medical history was LMS. She had taken Ifosfamide and mesna in her last chemotherapy. Seventy percent of her liver and her left kidney were removed 4 years ago to prevent the progress of the disease. Because of the increase in the level of creatinine and urea in the initial laboratory report, a Shaldon catheter was inserted for the patient, and she was under emergency dialysis for 3 h. In addition, during the six‐day hospitalization period, dialysis was done two times. Finally, the patient was discharged with improved clinical tests accompanied by a twice‐weekly dialysis order.

**Conclusion:**

Ifosfamide is metabolized into chloroacetaldehyde, which can cause acute kidney injury. Recovery from acute kidney injury may not always be perfect and can lead to some degree of chronic kidney disease. Opposite to hemorrhagic cystitis, mesna is not effective in preventing ifosfamide's nephrotoxicity and N‐acetylcysteine may be effective in the prevention of this nephrotoxicity.

## 1 INTRODUCTION

Leiomyosarcoma (LMS) is an aggressive soft tissue sarcoma derived from smooth muscles. LMS can occur in any soft tissue, but the uterus, stomach, arms, legs, and small intestine are the most common sites of this cancer.^1^

Uterine LMS is a rare malignancy that is aggressive and associated with a high risk of recurrence or death. A history of pelvic radiation, long‐term use of tamoxifen and hereditary conditions appear to be associated with an increased risk for the development of some LMS types.^2^ Hereditary leiomyomatosis and renal cell cancer (HLRCC) is a hereditary condition associated with the development of multiple LMS in the skin and uterus as well as an aggressive form of renal cancer. HLRCC is an autosomal dominant syndrome caused by mutations in fumarate hydratase.^3^

Most people who get this type of cancer are over 50 years old. Symptoms of LMS may vary from case to case depending upon the exact location, size, and progression of the tumor. Abdominal pain, abdominal distension, and premenopausal or postmenopausal abnormal uterine bleeding are the most common symptoms of LMS.^4^

Ifosfamide, cisplatin, and doxorubicin are the most chemotherapy regimens that are used for advanced metastatic LMS.^5^ Ifosfamide is an alkylating agent used for bladder cancer, cervical cancer, soft tissue sarcoma, testicular cancer, thymomas, thymic cancers, and some forms of lymphoma. Major adverse effects of Ifosfamide are hematuria, metabolic acidosis, and renal insufficiency. Other renal toxicities include tubulopathies such as proximal tubular injury or Fanconi syndrome and nephrogenic diabetes insipidus.^6^, ^7^

## 2 CASE PRESENTATION

This case report was conducted according to the Declaration of Helsinki principles. A‐44 year‐ old woman with the chief complaint of pain in the epigastric area, itching, coughing, nausea, and vomiting was referred to the emergency department of Imam Khomeini hospital located in Sari, North of Iran. Her medical history was positive in terms of the LMS disease that had been first diagnosed 7 years ago in her left kidney, which metastasized to the liver. She received 12 cycles of chemotherapy with doxorubicin (20 mg/m^2^/day for 3 days), ifosfamide (1500 mg/m^2^/day, for 2 days) and mesna (900 mg/m^2^/day with ifosfamide).

Her last chemotherapy was 16 days before this presentation. Seventy percent of her liver was removed through surgery 4 years ago due to hepatic metastasis. In addition, a hysterectomy was done, her left kidney was removed to prevent the progress of the disease, and now she is a single kidney patient.

**FIGURE 1:**
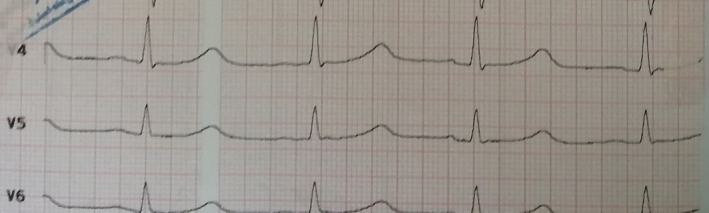
Bradycardia and QT interval prolongation (because of hyperkalemia).

Her vital signs immediately after entering the emergency department were as follow: Blood pressure: 128/80 mmHg, temperature: 37°C, heart rate: 98 beats/min, and respiratory rate: 13 breaths per minute. Her skin and sclera were icteric during the physical examination. She had poor oral tolerance. She took no medicine before admission. There is no history of smoking, tobacco, and alcohol use. In addition, she is not allergic to a specific food or medicine. During the routine procedures in the emergency department, ECG was taken from the patient and showed bradycardia (about 50 heartbeats/min) and QT interval prolongation (Figure 1).

Laboratory parameters at admission to the emergency department were presented in Table 1. According to the hyperkalemia in the preliminary tests, a kayexalate powder with lactulose was prescribed. Because of the increase in the level of creatinine and urea in the initial laboratory report in the emergency room, a nephrology consultation was requested. Based on the nephrologist's comments, the HbsAg and HCVAb tests were requested, and then the patient was hydrated with normal saline. According to the increase in the level of creatinine, serum potassium >6.0 mEq/L (6.3 mEq/L) and increase in LDH and ALP at the same time, the patient had indications for elective dialysis (it should be noted that, in the latest clinical laboratory data, our patient had normal creatinine levels and acceptable renal function). So a Shaldon catheter was inserted for the patient, and she was under elective dialysis for 3 h. She received a unit of packed cells during dialysis.

**TABLE 1:** Initial laboratory data in the emergency department.

Lab data parameter	Normal range	Result
WBC	4000–1000/mm^3^	9900/mm^3^
PLT	145 000–450 000/mm^3^	140 000/mm^3^
Hb	12.3–15.3 g/dl	**5.5 g/dl**
Blood sugar	90–110 mg/dl	136 mg/dl
Urea	13–40 mg/dl	**269 mg/dl**
Creatinine	0.5–1.3 mg/dl	**9.8 mg/dl**
AST	5–40 U/L	**87 U/L**
ALT	5–40 U/L	**60 U/L**
ALP	64–306 U/L	**1946 U/L**
Bil.T	0.2–1.2 mg/dl	**8.83 mg/dl**
Bil.D	0–0.3 mg/dl	**4.78 mg/dl**
K	3.5–5.5 mEq/L	**6.3 mEq/L**
Na	135–145 mEq/L	**128 mEq/L**
Calcium	8.5–10.5 mg/dl	8.3 mg/dl
Albumin	3.5–5.5 g/dl	3.7 g/dl
Mg	1.8–2.2 mg/dl	**3.9 mg/dl**
P	2.5–4.5 mg/dl	4.5 mg/dl
C.R.P	Less than 6 mg/L	**178 mg/L**
ESR	0–20 mm/h	**90 mm/h**
LDH	140–280 U/L	**1013 U/L**
Amylase	Less than 100 U/L	**368 U/L**
Lipase	Less than 60 U/L	**392 U/L**
pH	7.35–7.45	**7.25**
HCO_3_	22–28 mmol/L	**11.5 mmol/L**
PCO_2_	35–45 mmHg	**27.5 mmHg**
Troponin Q	Less than 100 ng/dl	12 ng/dL

Abbreviations: ALP, alkaline phosphatase; ALT, alanine transaminase; AST, aspartate aminotransferase; Bil.D, direct bilirubin; Bil.T, total bilirubin; CRP, C‐reactive protein; ESR, erythrocyte sedimentation rate; Hb, hemoglobin; K, potassium; LDH, lactic acid dehydrogenase; Mg, magnesium; Na, sodium; P, phosphorus; pH, power of hydrogen; PLT, platelet count; WBC, white blood cells.

Bold cases indicated abnormal patient laboratory data (relative to the reference range).

In addition, a urine sample was sent for analysis and culture at the beginning of the patient's admission. According to the results, no evidence of infection was observed in the administrative analysis.

Ondansetron was used to control nausea and vomiting. Heparin and pantoprazole were used as deep vein thrombosis (DVT) prophylaxis and prophylaxis for peptic ulcers, respectively. In addition, cetirizine was prescribed to control itching. The patient was transferred to the oncology ward after relative improvement in clinical status and laboratory parameters.

**FIGURE 2:**
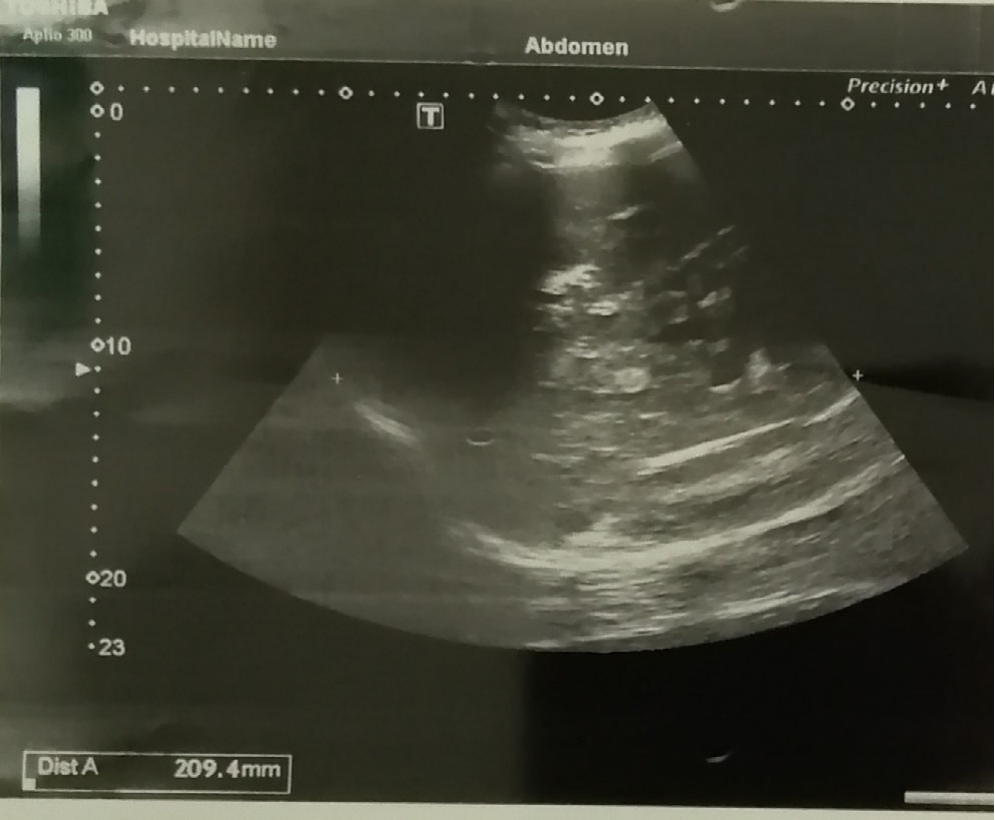
Abdominal ultrasonography.

In the abdominal and pelvic ultrasonography, the left kidney was not seen (removed 4 years ago to prevent the progress of the disease). The right kidney size was 126 mm, and a relatively increased parenchymal echo without any stones or hydronephrosis. In addition, the uterus and ovaries were not seen in their anatomical place (due to hysterectomy). The size of the liver was larger than the normal size. Isoechoic and hypoechoic lesions and echogenicity were seen all over the liver's lobes and no evidence in favor of the new metastasis was found (Figure 2).

During the hospitalization period in the oncology section, dialysis was done two times. In addition to the dialysis, the nephrologist recommended receiving calcium carbonate to control hyperphosphatemia.

Finally, the patient was discharged with improved clinical tests (Table 2) accompanied by a twice‐weekly dialysis order. According to the high level of phosphor and calcium, and also multiplying these two is more than 50, it was recommended to take sevelamer 800 mg twice daily (immediately after a meal) instead of calcium carbonate by the clinical pharmacy service.

**TABLE 2:** Laboratory data in the oncology ward

Lab data parameter (normal range)	Day 1^a^	Day 2	Day 3	Day 4	Day 5	Day 6
WBC (4000–1100/mm^3^)	10 800	7100	5000	5200	5100	4100
PLT (145 000–450 000/mm^3^)	140 000	**138 000**	**131 000**	**95 000**	**102 000**	90 000
HB (12.3–15.3 g/dl)	**5.0**	**8.9**	**7.8**	**7.1**	**6.9**	7.8
Urea (13–40 mg/dl)	**110**	**201**	**146**	**163**	**185**	121
Creatinine (0.5–1.3 mg/dl)	**7.5**	**4.7**	**3.7**	**4.1**	**4.5**	3.4
AST (5–40 U/L)	57	85	88	81	87	100
ALT (5–40 U/L)	53	53	55	51	60	18
ALP (64–306 U/L)	**1732**	**2038**	**2156**	**1879**	**1946**	2974
Bil.T (0.2–1.2 mg/dl)	**7.7**	–	**9.08**	**9**	**8.83**	–
Bil.D (0–0.3 mg/dl)	**4.9**	–	**4.85**	**4.82**	**4.78**	–
K (3.5–5.5 mEq/L)	**5.5**	4	3.6	3.6	3.7	3.5
Na (135–145 mEq/L)	131	133	140	136	135	133
Calcium (8.5–10.5 mg/dl)	–	–	–	–	8.5	–
Albumin (3.5–5.5 g/dl)	–	–	–	–	3.1	–
Mg (1.8–2.2 mg/dl)	–	2.55	–	–	–	–
P (2.5–4.5 mg/dl)	–	4.5	–	–	5.6	–
Amylase (less than 100 U/L)	**156**	**194**	**95**	–	**368**	–
Lipase (less than 60 U/L)	**541**	**179**	**72**	–	**392**	–

Abbreviations: ALP, alkaline phosphatase; ALT, alanine transaminase; AST, aspartate aminotransferase; Bil.D, direct bilirubin; Bil.T, total bilirubin; Hb, hemoglobin; K, potassium; Mg, magnesium; Na, sodium; P, phosphorus; PLT, platelet count; WBC, white blood cells.

^a^ Day1: First day of admission to the oncology ward.

Bold cases indicated abnormal patient laboratory data (relative to the reference range).

## 3 DISCUSSION

We reported acute kidney injury (AKI) in a patient with metastatic LMS that was previously treated with an ifosfamide‐based chemotherapy regimen. Ifosfamide causes hemorrhagic cystitis. However, there are limited reports of renal toxicity with this drug. Ifosfamide is a cytotoxic isomer of cyclophosphamide that requires biotransformation to become activated. The 4‐hydroxy‐ifosfamide, is a major metabolite of ifosfamide. The 4‐hydroxy‐ifosfamide is an active metabolite of ifosfamide and the anti‐cancer effects of ifosfamide have been attributed to this metabolite.^8^

Many drugs may cause pre‐renal or renal kidney injury. To distinguish pre‐renal acute kidney injury from renal acute kidney injury (intrinsic), measuring the BUN/ serum creatinine ratio is helpful. The BUN/serum creatinine values greater than 20:1 usually represent pre‐renal disease. This ratio is usually less than 15 in intrinsic acute kidney injury.^9^ In our patient, the BUN/serum creatinine ratio was 13.7, which rules out the existence of pre‐renal conditions.

Hyperkalemia, metabolic acidosis, hyperphosphatemia, hypocalcemia, and elevated serum CPK are the most frequent laboratory findings in AKI. As demonstrated in Table 1 and Table 2, many aspects of laboratory data of our patient were compatible with AKI including an increase in serum creatinine and uremia, hypermagnesemia, hyperkalemia, hyponatremia, and metabolic acidosis.

On the other hand, our patient has elevated levels of ESR, CRP, LDH, amylase, and lipase. In addition to some diseases such as pancreatitis, amylase and lipase also increase in AKI.^10^ In our patient, amylase was increased to threefold of the upper normal limit, and lipase was increased to six folds of the upper normal limit. In AKI, the amylase enzyme has been shown to increase up to 10 times the upper normal limit, while the lipase enzyme increase is usually less dramatic, reaching 3–5‐folds the upper normal limit.^11^ Furthermore, if these enzymes are elevated, using ultrasound to differentiate pancreatitis from other causes can be helpful. In our patient, there was no evidence in favor of pancreatitis on ultrasound imaging (Figure 2).

LDH levels are usually very high in AKI with widely different pathogenesis. Many studies demonstrated that the LDH levels significantly increased in AKI (especially rhabdomyolysis).^12^ CRP and ESR are two non‐specific inflammatory factors. CRP is not only an inflammation biomarker but also an activated mediator of AKI. The role of these biomarkers, especially in drug‐induced AKI, is prominent. It demonstrated the association between systemic inflammation biomarkers and local renal inflammation in drug‐induced tubulointerstitial nephritis.^13^

As in our patient, ifosfamide may cause tubular nephrotoxicity. In addition to the main pathway of Ifosfamide metabolism, it penetrates into tubular cells through human organic cation transporter II. In the tubular cells, ifosfamide is metabolized into nitrogen mustard (finally product acrolein) and chloroacetaldehyde (CAA). Toxic manifestations of these two metabolites are different. The acrolein metabolite appears to be responsible for hemorrhagic cystitis and the chloroacetaldehyde metabolite may be associated with nephrotoxicity and central nervous system toxicity.^14^

The main ifosfamide nephrotoxicity is tubular dysfunctions and signs such as proteinuria, glucosuria, polyuria, aminoaciduria, normal anion gap metabolic acidosis, renal tubular acidosis, and hypophosphatemia. Fanconi syndrome is the most reported nephrotoxicity with ifosfamide.^15^, ^16^, ^17^ Reduction in glomerular filtration rate (GFR) due to tubular dysfunctions, is usually mild, unless ifosfamide is given in high doses or in combination with other nephrotoxic drugs.^18^

In some research, unilateral nephrectomy, previous cisplatin treatment, and cumulative dose were important risk factors for ifosfamide nephrotoxicity.^19^ Table 3 demonstrated some clinical case reports and factors associated with ifosfamide‐induced AKI. Our patient received a cumulative ifosfamide dose of 36 g/m^2^, however, she had one kidney.

**TABLE 3:** Some case reports and clinical characteristics of patients with ifosfamide‐induced nephrotoxicity

Study	Type of cancer	Age/sex	cumulative dose of ifosfamide (g/m^2^)	Out come	Risk factors for AKI	Ref
Sohail et al. (2021)	Synovial cell sarcoma	36/Female	7.5	Ifosfamide‐induced nephrogenic diabetes insipidus	Unknown risk factors	[20]
Aisyi et al. (2020)	Wilms tumor	3/Male	Not reported	Fanconi syndrome	Wilms tumor and Multiple Ifosfamide regime	[21]
Sokolova et al. (2017)	Non‐seminomatous germ cell tumor	21/Male	4	Delayed rhabdomyolysis after high‐dose chemotherapy	Paclitaxel, ifosfamide, carboplatin, and etoposide regimen	[22]
Matsuura et al. (2014)	Osteosarcoma	15/Male	69.7	Acute interstitial nephritis (Karyomegalic)	High cumulative dose and the combination of cisplatin	[23]
Kamran et al. (2014)	Rhabdomyosarcoma	26/Female	23.4	Fanconi syndrome and nephrogenic diabetes insipidus	Unknown risk factors	[24]
Lee et al. (2014)	Synovial sarcoma	25/Female	39	Fanconi syndrome	Possible effect of high dose	[15]
Kim et al. (2008)	Ovarian cancer	32/Female	10	Proximal Tubule Injury	Combination of cisplatin and ifosfamide	[25]
Hill et al. (2000)	Breast cancer	Three women with age 40–50	20–30	Tubulo‐interstitial nephritis	High‐dose chemotherapy	[26]

The cumulative dose of ifosfamide may be an important factor in the incidence of AKI. The effect of the cumulative dose has been discussed differently in previous case reports. Different limits of a safe dose were reported in previous studies (for example, dose greater than 45, 50, or 60 g/m^2^).^19^ To our knowledge, patients who have experienced nephrotoxicity with ifosfamidewere exposed to cumulative doses of ifosfamide ranging from 4 to 100 g/m^2^. Moreover, some thresholds that are reported, may depend upon the age, type of cancer, genetic background, or chemotherapy regimens.^8^ Based on the above findings, it is not possible to comment definitively on the role of cumulative dose in our patient.

Hydration, forced diuresis, and urine alkalization with sodium bicarbonate (NaHCO_3_) have been suggested to prevent the bladdercomplications associated with ifosfamide.

Mesna (2‐mercaptoethane sulfonate Na) is a synthetic sulfhydryl compound that has long been used to prevent and reduce the hemorrhagic cystitis toxicity caused by acrolein.^27^ The effect of mesna on reducing ifosfamide kidney injury remains unclear.^28^ Concomitant administration of mesna with ifosfamide, can decrease the incidence of hemorrhagic cystitis. However, it does not prevent nephrotoxicity.^29^ Mesna is more effective for toxicity mediated by acrolein, and it did not prevent nephrotoxicity induced by chloroacetaldehyde. There are some case reports of nephrotoxicity, (especially Fanconi syndrome), even despite receiving mesna with ifosfamide.

The mechanism of ifosfamide‐induced nephrotoxicity may be related to oxidative damage. Glutathione and thiol groups are important anti‐oxidant substances in the renal system. Chloroacetaldehyde directly reacts with thiols groups, leading to a decrease in the levels of glutathione (GSH) in the tubular cells, and mediates its nephrotoxicity.^14^, ^30^ In the in‐vitro study, Yaseen et.al demonstrated that chloroacetaldehyde altered lactate metabolism in isolated proximal tubules, and treatment with mesna, did not alter glutathione levels in renal cells.^31^

According to the mentioned mechanisms, the use of antioxidant drugs can be effective in preventing the toxicity of ifosfamide. N‐acetylcysteine (NAC) has been effective in animal models and in vitro studies for the prevention of ifosfamide nephrotoxicity.^32^ The lowering effect of chloroacetaldehyde on intracellular GSH can be prevented by NAC. On the other hand, in vitro research shows that NAC treatment significantly maintains the effective level of GSH in renal tubular cells.^33^

In addition, in some reports, it has been suggested that urine acidification can reduce the lowering effect of chloroacetaldehyde on intracellular GSH. Therefore, urinary acidification can be considered an option to prevent ifosfamide nephrotoxicity.^34^, ^35^

## 4 CONCLUSION

Ifosfamide is an alkylating agent that is used for bladder cancer, cervical cancer, soft tissue sarcoma, and some lymphoma. Chloroacetylaldehyde and acrolein are two major active metabolites of Ifosfamide. Chloroacetylaldehyde can cause acute kidney injury with tubular damage. Recovery from ifosfamide acute kidney injury may not always be complete and can leading to some degree of chronic kidney disease. Opposite to hemorrhagic cystitis, mesna is not effective in preventing the other aspects of ifosfamide's nephrotoxicity. Some research has shown that NAC with anti‐oxidant activity may prevent the nephrotoxicity of ifosfamide. However, extensive clinical trials are needed to confirm the efficacy of NAC. Therefore, the use of NAC can be considered a potential preventive factor for future studies.

## AUTHOR CONTRIBUTIONS

Ebrahim Salehifar was involved in editing and preparing the final version of the manuscript. All the authors reviewed the paper and approved the final version of the manuscript.

## CONFLICT OF INTEREST

The authors have stated explicitly that there are no conflicts of interest in connection with this article.

## ETHICS STATEMENT

The study was approved by the local ethics committee.

## Data Availability

The data are available with the corresponding author and can be achieved on request.
